# Supplementary material for: A Phase II Study of Denileukin Diftitox in Patients with Advanced Treatment Refractory Breast Cancer
Source: Vaccines (Basel). 2025 Jan 24;13(2):117. doi: 10.3390/vaccines13020117 (PMC11860294; doi:10.3390/vaccines13020117)
Supplement: Supplementary file 1 [file vaccines-13-00117-s001.zip › vaccines-3294419-supplementary.pdf]

Supplementary Table S1. Detailed adverse events.

All Adverse Events (n=589)	Unrelated		Related			
	Grade 1	Grade 2	Grade 1	Grade 2	Grade 3	Grade 4
**Auditory/Ear**						
Other - Ringing		1				
Tinnitus			1			
**Blood/Bone Marrow**						
ANC/AGC abnormal	1	1	1			
Hemoglobin abnormal	11	2	2	2	1	
Leukocytes abnormal			6	1		
Lymphopenia				2	5	1
Other - HCT low			1	1		
Other - MCV and MCH high	1					
Other - RBC low			2	1		
Platelets abnormal	1		3	1	1	
**Cardiac Arrhythmia**						
Other	2					
**Cardiac General**						
Hypertension			2			
Hypotension			2	2		
**Constitutional Symptoms**						
Fatigue			23	8	5	
Fever (without neutropenia)			5			
Insomnia	7		7			
Rigors/chills	1		11			
Weight gain			1	1		
Weight loss	1					
**Dermatology/Skin**						
Flushing		1				
Rash/desquamation	1		1			
Rash: acne/acneiform		1				
**Gastrointestinal**						
Anorexia	7	1	2	4		
Constipation	10	4	6	6		
Dehydration		2				
Diarrhea	2		2		1	
Gastritis	2					
Nausea	5	2	10	4	1	
Taste alteration (dysgeusia)	1					
Vomiting	3	1	2	3	1	
**Infection**						
Other - Toe	1					
**Lymphatics**						
Edema - limb			5	1		

All Adverse Events (n=589)	Unrelated		Related			
	Grade 1	Grade 2	Grade 1	Grade 2	Grade 3	Grade 4
**Metabolic/Laboratory**						
Alkaline phosphatase abnormal	3	1	5	2		
Alkalosis (metabolic or respiratory)			1			
ALT abnormal	6	5	12	8	2	
AST abnormal	9	6	13	6		
Creatinine abnormal	12	2	5			
Hyperbilirubinemia			1			
Hyperglycemia	14	3	3	1		
Hypoalbuminemia			18	9		
Hypocalcemia	4	3	18	3		
Hypoglycemia	2		2			
Hypokalemia	5		24		2	
Hyponatremia			2			
Hypoproteinemia			7			
Other - Chloride high			5			
**Musculoskeletal/Soft Tissue**						
Muscle weakness				1	1	
Other - Leg cramps			1			
**Neurology**						
Ataxia (incoordination)	1					
Confusion	1		1			
Dizziness	1	1	1	1	1	
Mood alteration	3					
Neuropathy - motor	3	1				
Neuropathy - sensory	2	1	1			
**Ocular/Visual**						
Other - Loss of acuity related to illness		1				
Other - Vitreous detachment	1					
Vision-blurred vision			1			
Watery eye	1					
**Pain**						
Abdomen NOS	2	1				
Arthralgia					1	
Back	4	1	1	1		
Bone	2	1				
Extremity	1	1				
Head/headache	9		8	1		
Joint	3	1	2			
Muscle			3			
Myalgia	5	1	2			
Other - Arm		1				
Other - Musculoskeletal - Extremity		1				
Other - Pulmonary/Upper Respiratory - Chest Wall	4					
Other - Rib Pain	1					
Other - Tightness in Shoulders Secondary to Benadryl IV		1				
Throat/pharynx/larynx	1					
**Pulmonary/Upper Respiratory**						
Cough	8	2	1	1		
Dyspnea (shortness of breath)	5	2	3	1		
Other - Rhinitis	1	1		1		
Pleural effusion (non-malignant)				1		
Pulmonary fibrosis (radiographic changes)	1					
**Syndromes**						
Flu-like syndrome	2		6	2		
**Vascular**						
Acute vascular leak syndrome				11	8	
**Allergy/Immunology**						
Allergic reaction/hypersensitivity (including drug fever)		1				
